# A Wilkinson power divider with harmonic suppression through low-pass filter for GSM and LTE applications

**DOI:** 10.1038/s41598-024-52506-5

**Published:** 2024-01-29

**Authors:** Nariman Mohammadi, Gholamhosein Moloudian, Saeed Roshani, Sobhan Roshani, Fariborz Parandin, Ali Lalbakhsh

**Affiliations:** 1 Electrical Engineering Department, Salman Farsi University, Kazerun, Iran; 2 Tyndall National Institute, University College Cork, Cork, T12R5CP Ireland; 3 Department of Electrical Engineering, Kermanshah Branch, Islamic Azad University, Kermanshah, Iran; 4 Macquarie University College, Sydney, Australia

**Keywords:** Electrical and electronic engineering, Electronics, photonics and device physics

## Abstract

Conventional Wilkinson power dividers (WPDs) perform satisfactorily near the intended operation frequency. Nonetheless, these WPDs demonstrate subpar performance in the stopband and necessitate a significant physical space. To enhance the existing level of advancement and in order to improve on the current state-of-the-art, a modified WPD is designed and fabricated, demonstrating a significant improvement in stopband and superior isolation between output ports. To improve the stopband and suppress unwanted harmonics, a low-pass filter (LPF) structure is placed in the both branches of the conventional WPD. The proposed modified WPD depicts a wide stopband bandwidth (*f*_SB_ > 17.25 GHz) from 2.75 to over 20 GHz with an attenuation level of 20 dB, suppressing 2nd to 11th harmonics**.** According to measured results, the input return loss (|S_11_|), insertion loss (|S_21_|) and output isolation (|S_32_|) at *f* = 1.8 GHz are better than 33 dB, 3.2 dB and 21 dB, respectively. Indeed, the proposed modified WPD exhibits a magnitude imbalance of 0.00018, a phase imbalance of 1.25 degrees and a group delay of 0.5 ns. The proposed WPD depicts a compact size of 35 mm × 25 mm (0.38 λg × 0.27 λg), where λg is the guided wavelength at *f* = 1.8 GHz. There is a good agreement between the simulated and measured results. According to the obtained results, the proposed modified WPD shows a desirable performance for modern LTE and GSM communication applications.

## Introduction

With the rapid growth of technology in recent decades, the need to expand and upgrade wireless communication devices has increased dramatically. According to modern comprehensive needs to improve quality and the need for higher speed in wireless communication, special attention has been paid to increasing the quality and upgrading active and passive microwave devices such as antennas^1–5^, filters^6–18^, multiplexers^19–24^, and power dividers^25–44^. Power divider plays a vital role in modern microwave devices. The function of these devices is like a bridge between different parts of microwave circuits, making it crucial to pay special attention to the design of these devices. The most important features of a suitable power divider are low transmission loss (|S_31_| or |S_21_|), low input return loss (|S_11_|), low output return loss (|S_22_| or |S_33_|), high isolation between output ports (|S_23_|), low phase difference between output ports (phase imbalance), easy manufacturing, and compact size.

Microstrip filters such as bandpass filters (BPFs)^6,7^ and lowpass filters (LPFs)^8–18^ have a key role in microwave circuits to suppress unwanted harmonics. In recent years, several techniques and topologies such as defected ground structure (DGS)^8–10^, T-shaped resonators^11,12^, microstrip stubs^13–15^, and hexagonal-shaped resonators^16–18^ have been reported for designing LPF structures. According to the current state-of-the-art, design and fabrication of an LPF structure with sharp roll-of-rate (ROR), high suppression factor (SF), high relative stopband (RSB), high figure of merit (FOM), compact size and affordable manufacturing processes are still design challenges^15^. A tunable microstrip LPF structure with sharp ROR and wide stopband bandwidth has been employed in an envelope detector/rectifier circuit for controlling harmonics^18^.

The demand for multi-port components, such as diplexers^19–24^ and power dividers^25–44^ has been experiencing significant growth in contemporary communication systems. Indeed, by integrating filters with power dividers and diplexers, more desirable features such as increasing the stopband, improving suppression bandwidth to eliminate unwanted harmonics and reducing the size and footprint can be achieved. A rat-race coupler with an LPF structure, compact size and high isolation was presented in^25^. Recently, several techniques and topologies for designing filtering power dividers, such as squared resonator based on artificial intelligence^26,27^, microstrip open and short stubs^28^, high/low impedance ring resonators^29,40,42^, open complementary split-ring resonator^30^, capacitor loading^31^, square-loop resonator and meandered stubs^32^, parallel strip line and DGS^34^, hexagonal shaped resonator and multi open stubs^35^, radial resonator and multi open stubs^36,37^ and microstrip electromagnetic bandgap element^43^ have been reported. However, a modified WPD with LPF structure and high isolation has been presented in^44^, the footprint is a bit large. In the cutting-edge next-generation communications, the design of a WPD that offers miniaturisation, harmonic suppression, high isolation, wide stopband performance, sharp ROR, high SF, and low-cost implementation remains a challenge at present.

In this paper, a WPD with a novel LPF harmonic structure is designed, fabricated and tested. The contributions of this work are (i). designing a modified WPD that can work at GSM and LTE frequency bands (*f* = 1.8 GHz), (ii) developing a novel LPF structure step by step based on theoretical equations and LC circuits, (iii) manipulating and controlling unwanted harmonics by employing the LPF structure into both branches of the WPD, and (iv) implementing a modified WPD with high isolation, low insertion loss and compact size in comparison with the current state-of-the-art. The rest of this paper is organised as follows. In Section "Materials and methods", the proposed WPD (conventional WPD, LPF structure, and modified WPD) are designed and manufactured. We undertake the performance evaluation of the fabricated circuit and discuss simulated and measured results in Section "Results and Discussion". Finally, Section "Conclusions" concludes this paper.

## Materials and methods

### Power divider structure design

**Figure 1:**
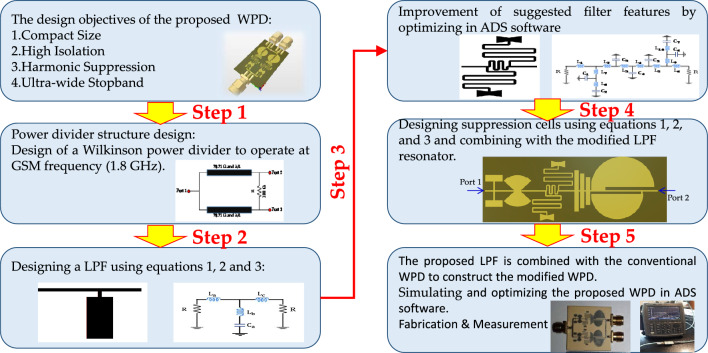
The design procedure of the proposed WPD.

The WPD design methodology is depicted in Fig. 1. The aim is to design a modified WPD with a compact size, high isolation, ultra-wide rejection band and high suppression factor. In the first step, a conventional WPD is designed and simulated. A basic LC resonator is designed based on normalisation coefficients of the third-order LPF circuit and mathematical analysis^45^ in the second step. The roll-off-rate (ROR) is one of the important performance parameters for the LPF structures, which shows the changing rate from the passband to the stopband. A novel resonator is designed to improve the LPF response (sharpen ROR parameter) compared to the basic resonator. A novel harmonic suppression is presented and analysed in the fourth design step. The proposed novel harmonic suppression is used to achieve a wide stopband and suppress unwanted harmonics. Then, the novel harmonic suppression structure is added to the proposed novel modified resonator to create an LPF structure. In the final step, the proposed LPF structure is replaced in the both branches of conventional WPD to pass DC frequency signal and fundamental harmonic and eliminate other harmonics (2nd to 11th harmonics). Placing filters within the branches of a WPD can offer several advantages, but it is important to justify this design choice based on specific requirements and constraints. Several potential advantages and justifications of employing LPF structures in the WPD branches are size mitigation, reducing signal path length and improving isolation between output ports. However, it is important to note that the choice to integrate filters within the power divider branches should be made after considering the specific requirements of the application, including frequency range, desired filter characteristics, size constraints, and performance goals.

**Figure 2:**
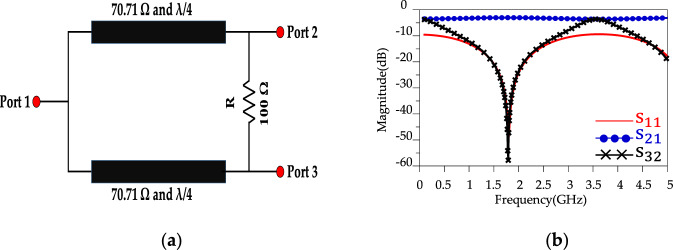
Conventional WPD. (**a**) Schematic and (**b**) simulation results.

**Figure 3:**
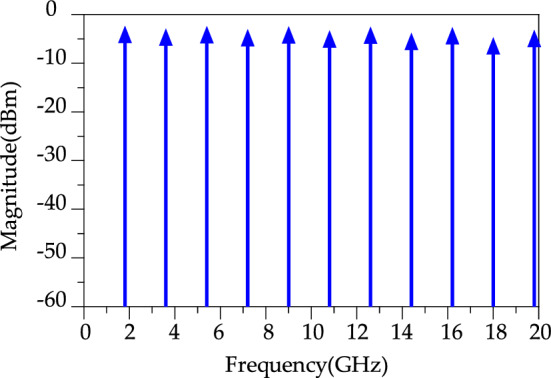
Simulated output harmonic balance.

A conventional WPD is designed with two-quarter wavelength (λ/4) microstrip transmission lines with the impedance of each branch 70.71 Ω (${Z}_{0}\sqrt{2} and {Z}_{0}=50\Omega$), and a resistance of 100 Ω (R = 2 ${Z}_{0}\Omega )$ which is placed between the two output ports. A schematic for the block diagram and simulation results of the proposed conventional WPD at *f* = 1.8 GHz is shown in Fig. 2a. According to Fig. 2b, the proposed conventional WPD shows a well performance for the input return loss (S_11_), isolation (S_32_) and insertion loss (S_21_) at *f* = 1.8 GHz. Figure 3, depicts a high-frequency harmonic balance analysis from 0 to 20 GHz. According to Fig. 3, the magnitude of the harmonic balance is very close to -3 dBm for an input power *P*_IN_ = 0 dBm. In the following, an LPF structure will be designed in order to improve the WPD features such as suppressing unwanted harmonics, high isolation, wider stopband bandwidth and compact size.

### LPF design methodology

In the first stage of LPF design, a simple LPF resonator is designed based on the normalisation coefficients of the third-order LPF circuit and mathematical analysis^45^.

1.a $${L}_{i}=\frac{{Z}_{0}{g}_{Li}}{2\pi {f}_{c}}$$

1.b $${C}_{i}=\frac{{g}_{Ci}}{2\pi {f}_{c}{Z}_{0}}$$

**Figure 4:**
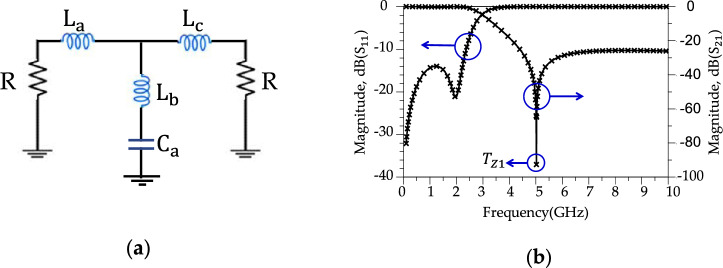
The LPF resonator. (**a**) Schematic circuit. (**b**) Simulation results.

In the above Eqs. (1.a) and (1.b), ${Z}_{0}$ is the characteristic impedance of the transmission line, which is equal to 50 Ω. *f*_c_ is a − 3 dB cut-off frequency, ${g}_{Ci}$ and ${g}_{Li}$ are the normalisation coefficients of the LPF circuit. From^45^, the normalisation coefficients of the LPF circuit are ${g}_{L1}$ = 1.130, ${g}_{L2}$ = 0.2559, ${g}_{C2}$ = 1.138, and ${g}_{L3}$ = 1.360. Using Eqs. (1.a) and (1.b), an LPF resonator is designed for *f*_c_ = 2.7 GHz. In Fig. 4, the circuit diagram of the designed filter can be seen along with the shape of its response. In Fig. 4a, the value of L_a_ = 3.3 nH, L_b_ = 0.75 nH, L_c_ = 4 nH and C_a_ = 1.34 pF. According to Fig. 4b, the transmission zero (${T}_{Z}$) can be calculated by Eq. (2). In Fig. 4b, |*T*_z1_ |= 92 dB (at *f*_Tz1_ = 5.02 GHz).

2 $${T}_{z}=\frac{1}{2\pi \sqrt{{C}_{a}{L}_{b}}}$$

In the following, using Eqs. (3.a) and (3.b), schematic circuits are converted into the layout structure. The values of the inductors and capacitors of the third-order circuit can be obtained using Eqs. (1.a) and (1.b). In addition, the physical lengths of the low- and high-impedance lines are calculated using Eqs. (3.a) and (3.b).

3.a $${l}_{{L}_{i}}=\frac{{\lambda }_{g{L}_{i}}}{2\pi }{{\text{sin}}}^{-1}(\frac{2\pi {f}_{c}{L}_{i}}{{Z}_{0L}})$$

3.b $${l}_{{C}_{i}}=\frac{{\lambda }_{g{C}_{i}}}{2\pi }{\mathit{sin}}^{-1}(2\pi {f}_{c}{C}_{i}{Z}_{0C})$$

**Figure 5:**
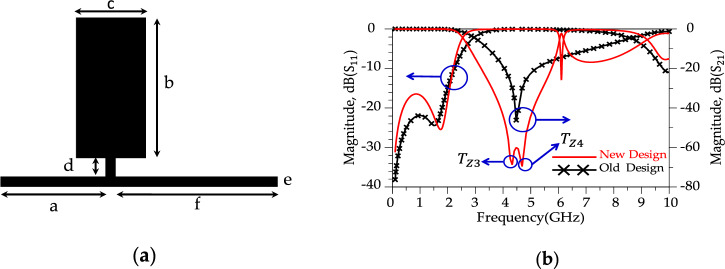
(**a**) Layout for the conventional resonator. (**b**) Simulated results.

In the Eqs. (3.a) and (3.b), ${\lambda }_{g{L}_{i}}$ and ${\lambda }_{g{C}_{i}}$ are the corresponding guided wavelengths, and ${Z}_{0C}$ and ${Z}_{0L}$ are the impedances of transmission lines with low and high impedance, respectively. Figure 5a, shows the layout designed using Eqs. (3.a) and (3.b). Figure 5b, shows the simulated results for the the conventional resonator. The dimension of the conventional resonator (depicted in Fig. 5a) are a = 5.9 mm, b = 7 mm, c = 3.5 mm, d = 1 mm, e = 0.5 mm and f = 7.5 mm.

**Figure 6:**
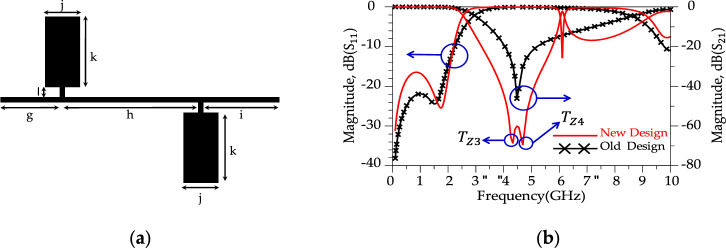
(**a**) Layout for the modified resonator #1. (**b**) Simulated results. (show T_Z3_ and T_Z4_).

According to Fig. 5b, the ROR is 13.65 dB/GHz for the conventional resonator and|*T*_z2_ |= 49 dB (at *f*_Tz1_= 4.534 GHz). To improve the ROR and achieve a sharp response, two conventional resonator are placed in series, shown in Fig. 6. The dimension for the modified resonator #1 (shown in Fig. 6a) are g = 5.9 mm, h = 13.2 mm, i = 7.5 mm, j = 3.5 mm, k = 7 mm and l = 1 mm.

**Figure 7:**
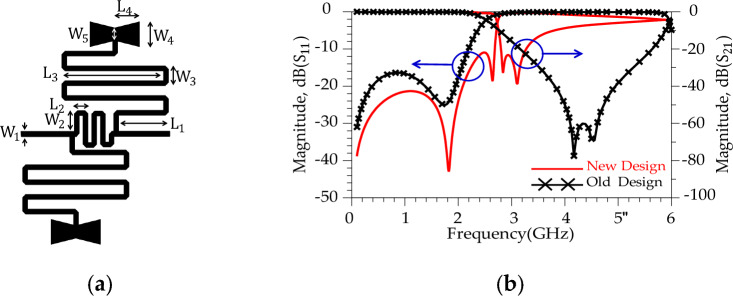
Proposed structure (modified resonator #2), (**a**) Layout. (**b**) Simulation results.

**Figure 8:**
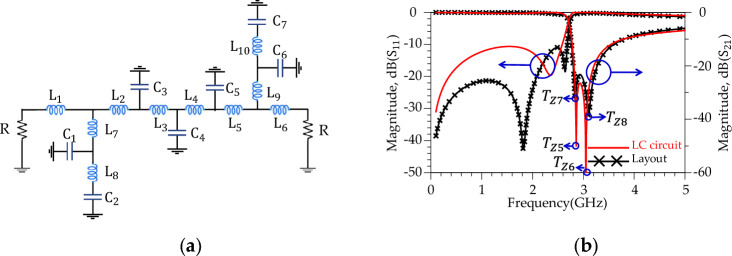
Proposed structure (**a**) Schematic of LC equivalent circuit. (**b**) Simulation results. The values of the inductors and capacitors are L_1_ = 1.5 nH, L_2_ = 1 nH, L_3_ = 1 nH, L_4_ = 1 nH, L_5_ = 1 nH, L_6_ = 1.5 nH, L_7_ = 6.8 nH, L_8_ = 2.7 nH, L_9_ = 6.8 nH, L_10_ = 1.3 nH, C_1_ = 0.05 pF, C_2_ = 0.3 pF, C_3_ = 0.1 pF, C_4_ = 0.1 pF, C_5_ = 0.1 pF, C_6_ = 0.05 pF and C_7_ = 0.3 pF.

Aiming for a higher Roll-of-rate (ROR), the conventional resonator was modified. These modifications manipulate the distributed surface current and electrical field and improves the frequency response of the conventional resonator^44^. The new design structure illustrates better response (sharper ROR and higher SF) than the previous structure. According to Fig. 6b, the new resonator (shown in Fig. 6a) shows a sharp ROR = 23.5 dB/GHz and two transmission zeros; |*T*_z3_|= 74 dB (at *f*_Tz3_= 4.02 GHz) and |*T*_z4_ |= 70 dB (at *f*_Tz4_= 4.583 GHz), respectively. In the following, a much better response (sharper ROR and more *T*_zs_) is obtained by making changes in the resonator structure. Figure 7 displays the suggested alteration in the structure of resonator #2 along with the corresponding simulated outcomes. As depicted in Figure 7b, the modified resonator #2 exhibits a more pronounced response when contrasted with modified resonator #1. Additionally, the return loss of modified resonator #2 displays an improved response and a broader resonance range compared to modified resonator #1. Furthermore, the overall size of modified resonator #2 has been notably decreased in comparison to the dimensions of modified resonator #1. This reduction in size not only lowers construction costs but also results in a smaller final design. According to the Fig. 8b, the modified resonator #2 illustrates a sharp ROR = 175 dB/GHz and four transmission zeros; |*T*_z5_ |= 50 dB (at *f*_Tz5_= 2.860 GHz), |*T*_z6_ |= 64.8 dB (at *f*_Tz6_= 3.05 GHz), |*T*_z7_ |= 32.75 dB (at *f*_Tz7_= 2.837 GHz) and |*T*_z8_ |= 39.278 dB (at *f*_Tz8_= 3.104 GHz), respectively. The dimension of the modified resonator #2 (depicted in Fig. 7), W_1_= 0.2 mm, W_2_ = 0.85 mm, W_3_ = 0.8 mm, W_4_ = 1 mm, W_5_ = 0.5 mm, L_1_ = 2 mm, L_2_ = 0.6 mm, L_3_ = 4.1 mm and L_4_ = 1 mm. The LC equivalent circuit for the proposed modified resonator #2 is shown in Fig. 8. To achieve an accurate LC equivalent circuit, the meandering lines in the layout of the modified resonator have been modelled by a π-type circuit according to the reference^44^. Apart from the modelling approach used here, there are other techniques based on modal analysis^46^, artificial intelligence-based approaches^47^ and transfer functions^48,49^. According to the obtained results, there is a good agreement between the simulated results for the layout and LC equivalent circuit.

**Figure 9:**
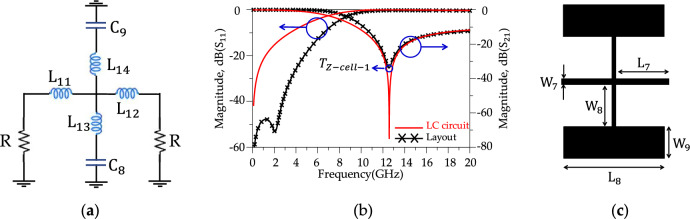
First suppression cell. (**a**) Schematic circuit. (**b**) Simulation results. (**c**) Layout.

**Figure 10:**
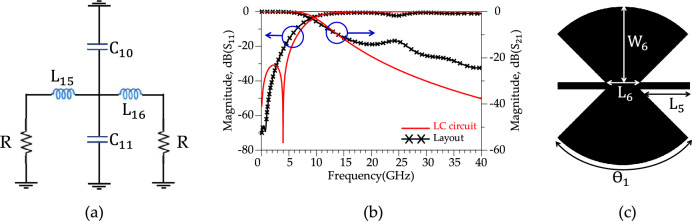
Second suppression cell. (**a**) Schematic circuit. (**b**) Simulation results. (**c**) Layout.

**Figure 11:**
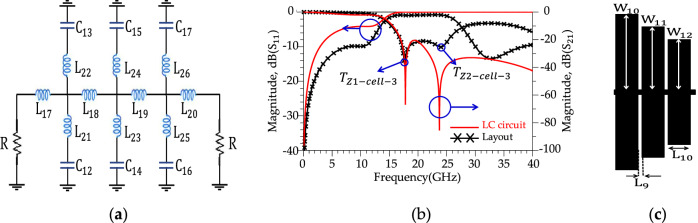
Third suppression cell. (**a**) Schematic circuit. (**b**) Simulation results. (**c**) Layout.

**Figure 12:**
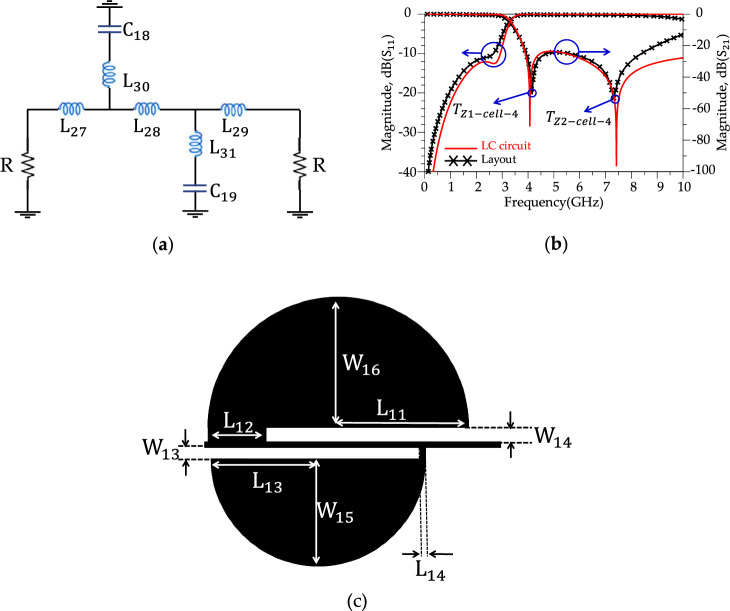
Fourth suppression cell. (**a**) Schematic circuit. (**b**) Simulation results. (**c**) Layou.

According to Fig. 8b, the proposed modified resonator illustrates a sharp response (ROR = 175 dB/GHz) and frequency selection (four *T*_zs_), but the stopband bandwidth is weak. Harmonic suppression structures to control unwanted frequencies are necessary to improve the rejection bandwidth. The proposed harmonic suppression structure consists of four suppression cells; each suppresses a frequency range in the rejection band. Fig. 9 shows the schematic circuit of the first suppression cell, simulated results and layout. According to Fig. 9b, the first suppression cell suppresses the S_21_ from 10.1 GHz to over 20 GHz with 10 dB attenuation level and creates a |*T*z-_Ce11-1_ |= 34 dB at *f*_*T*z-Ce11-1_ = 12.59 GHz. In Fig. 9a, the inductors and capacitors values are L_11_ = 0.2 nH, L_12_ = 0.5 nH, L_13_ = 0.4 nH, L_14_ = 0.4 nH, C_8_ = 0.4 pF and C_9_ = 0.4 pF. Moreover, In Fig. 9c, the dimensions of the first suppressor cell are L_7_ = 1.16 mm, L_8_ = 2.5 mm, W_7_ = 0.2 mm, W_8_ = 1.3 mm and W_9_ = 1 mm. Fig. 10, depicts the schematic circuit, simulated results and layout of the second suppressor cell. According to Fig. 10b, the second harmonic suppressor cell creates more than 10 dB attenuation levels from 13.78 GHz to over 20 GHz. The optimized LC circuit values are L_15_ = 0.9 nH, L_16_ = 0.9 nH, C_10_ = 0.3 pF and C_11_ = 0.3 pF. The dimensions of the second suppressor cell are L_5_ = 1.43 mm, L_6_ = 1 mm, W_6_ = 2.2 mm and ϴ = 90 degrees. According to the Fig. 11, the third harmonic suppressor cell suppresses the S_21_ in the stopband from 17.38 GHz to 28.5 GHz with 20 dB attenuation level and creates two transmission zeros;|*T*z_1_−_Ce11-3_ |= 34.37 dB at *f*_*T*z1-Ce11-3_ = 17.98 GHz and |*T*z_2_−_Ce11-3_ |= 29.75 dB at *f*_*T*z2−Ce11-3_ = 23.64 GHz. The optimised LC circuit values of the third suppressor cell are L_17_ = 1.57 nH, L_18_ = 0.5 nH, L_19_ = 0.5 nH, L_20_ = 0.05 nH, L_21_ = 1.6 nH, L_22_ = 1.6 nH, L_23_ = 0.9 nH, L_24_ = 0.9 nH, L_25_ = 0.24 nH, L_26_ = 0.24 nH, C_12_ = 0.05 pF, C_13_ = 0.05 pF, C_14_ = 0.05 pF, C_15_ = 0.05 pF, C_16_ = 0.05 pF and C_17_ = 0.05 pF. The physical length of the third harmonic suppressor cell are W_10_ = 3 mm, W_11_ = 2.5 mm, W_12_ = 2 mm, L_9_ = 0.62 mm and L_10_ = 0.21 mm. Moreover, according to Fig. 12, two radial stubs are employed to suppress lower frequency bands from 3.8 GHz to 9.1 GHz with 20 dB attenuation level as the fourth suppressing cell. In addition, according to Fig. 12b, the fourth suppressor cell creates two transmission zeros;|*T*z_1_−_Ce11-4_ |= 56.2 dB at *f*_*T*z1−Ce11-4_ = 4.15 GHz and|*T*z_2_−_Ce11-4_ |= 48 dB at *f*_*T*z2−Ce11-4_ = 7.58 GHz. The length and width values of the fourth suppressor cell are: L_27_ = 0.025 nH, L_28_ = 3.7 nH, L_29_ = 1.3 nH, L_30_ = 0.4 nH, L_31_ = 2.04 nH, C_18_ = 1.15 nH and C_19_ = 0.75 nH. The inductance and capacitance values for the LC circuit of the fourth suppressor cell were optimised to W_13_ = 0.39 mm, W_14_ = 0.48 mm, W_15_ = 3.7 mm, W_16_ = 4.5 mm, L_11_ = 4.5 mm, L_12_ = 2 mm, L_13_ = 3.7 mm and L_14_ = 0.2 mm.

**Figure 13:**
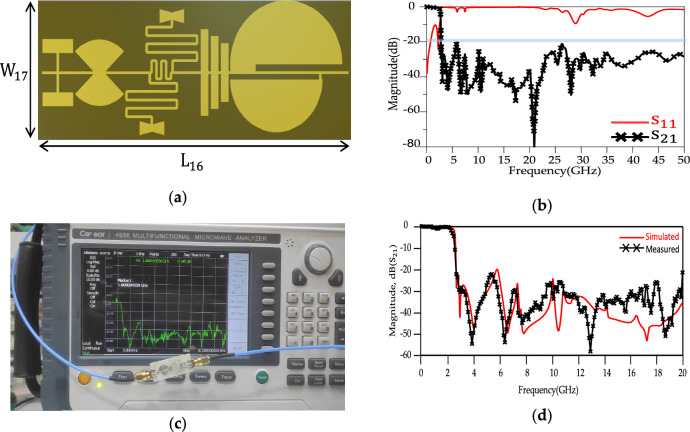
Proposed LPF (**a**) Layout. (**b**) Simulation results from 0 to 50 GHz. (**c**) Experimental setup. (**d**) S_21_ simulated and measured results up to 20 GHz. The values of L_16_ = 25 mm and W_17_ = 9.8 mm.

A suppression structure (four harmonic suppressor cells) have been added to the modified LPF resonator to achieve a large stopband and improve the frequency response in the rejection band. According to obtained results, good agreements are observed between the layout and LC circuit results. The introduction of the suppressor cells to the modified LPF resonator yields an LPF structure with a sharp response and large stopband as depicted in Fig. 13. The final structure of the LPF structure, simulated results and measured results are shown in Fig. 13. Table 1 shows a comparison between the simulated results for the proposed LPF structure and recent state-of-the-art. According to Fig. 13b, the—3 dB cut-off frequency for the proposed LPF structure is 2.69 GHz. The proposed LPF structure illustrates a sharp ROR parameter and equal to 180 dB/GHz. The return loss (RL) and insertion loss (IL) in the passband and stopband are better than 19, 0.53, 0.56 and 20 dB, respectively. The suppression level in the rejection band is better than 20 dB leading to SF parameter being 2. The proposed LPF structure shows an ultra-wide stopband bandwidth which covers a 17.31 GHz range (from 2.69 to over 20 GHz), with an RSB of 1.79 having been obtained.

**Table 1:** Comparison between simulated results and other studies for the LPF structure.

References	${{\varvec{f}}}_{{\varvec{c}}}$ (GHz)	ROR (dB/GHz)	RSB	SF	Size (${\lambda }_{g}^{2}$)	Size (mm)	IL (dB)
^9^	1	78	-	2	0.16 × 0.1	33.0*21.4	0.3
^10^	1.9	85	1.43	2.5	0.31 × 0.21	36.0*22.0	0.5
^11^	1.86	96.3	1.31	3.4	0.21 × 0.08	27.0*14.4	0.24
^12^	2.68	42.5	1.51	2	-	12.4*11.9	0.12
^13^	1.03	86.2	1.74	3	0.26 × 0.13	42.3*20.8	0.3
^16^	2.97	84.69	1.51	2	0.14 × 0.15	10.04*10.89	0.07
^17^	2.45	100		2	–	–	0.85
This work	2.695	180	1.795	2.0	0.412 × 0.161	25.0*9.80	0.53

The proposed structure has ROR and RSB better than previous works introduced in Table 1. Also, the dimensions of the structure are better than^9–11^.

**Figure 14:**
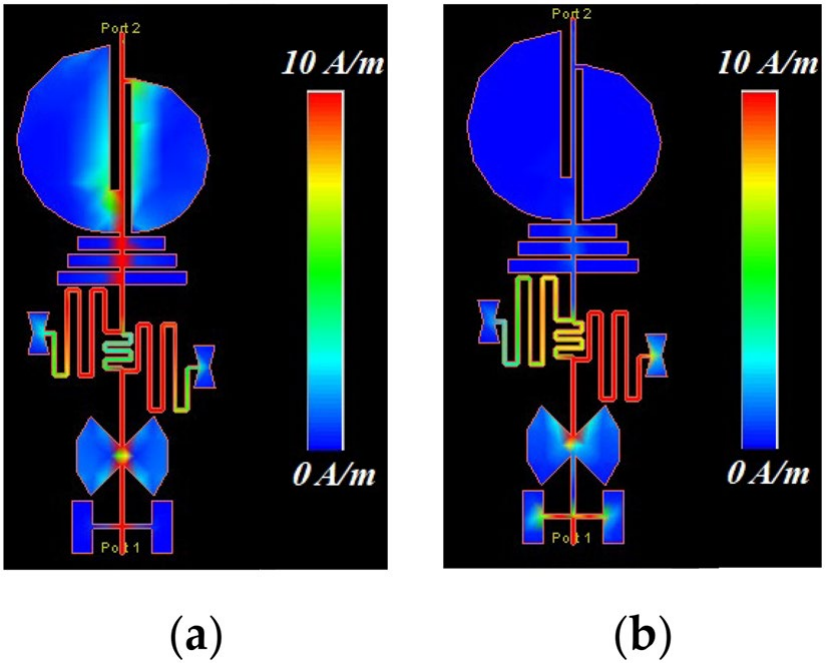
Surface current simulation (**a**) *f* = 1.8 GHz. (**b**) *f* = 3.17 GHz.

To validate the concept of the design methodology, the proposed LPF structure is subjected to simulation at two distinct frequency bands to showcase its performance in both the passband and the stopband. Fig. 14 illustrates the simulation results of the surface current distribution for the proposed LPF structure. As depicted in Fig. 14a, at a frequency of 1.8 GHz, which falls within the passband region of the LPF, the surface current flows towards port 2, clearly indicating the passband operation. In Fig. 14b, at a frequency of 3.17 GHz within the stopband, the LPF structure effectively blocks the signal and surface current.

### Proposed WPD

**Figure 15:**
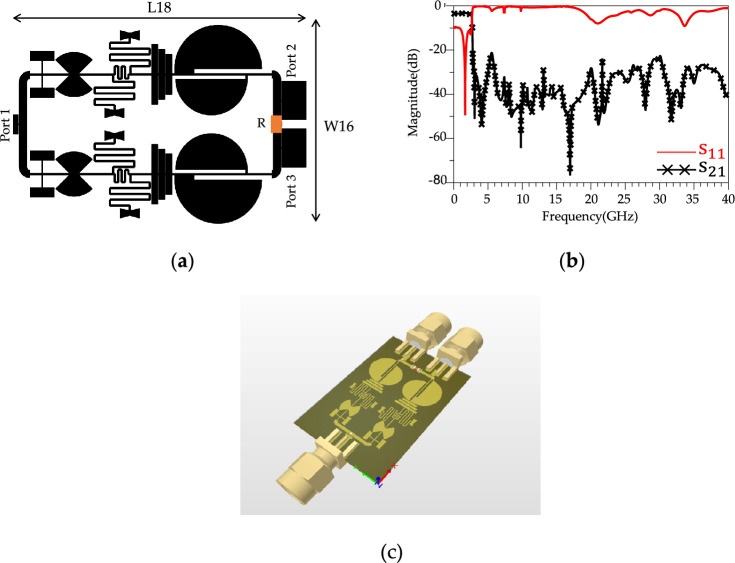
The proposed modified WPD. (**a**) Layout. (**b**) Simulation results. (**c**) 3D-Schematic. . The values of L_18_ = 35 mm, W_16_ = 25 mm and R = 100 Ω.

The proposed LPF structure is integrated into both branches of conventional WPD to improve the stopband bandwidth, suppress unwanted harmonics and increase the isolation between output ports. The schematic of the final layout of the proposed modified WPD and simulated results are depicted in Fig. 15.

**Figure 16:**
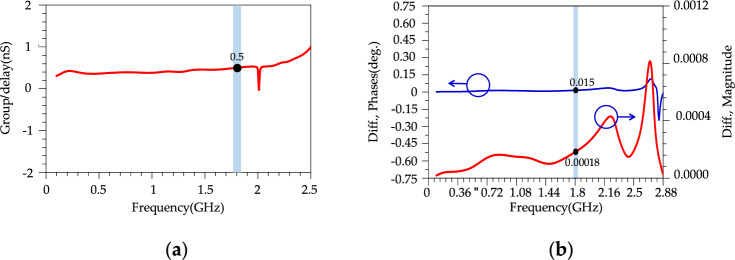
(**a**) Simulation results of Group delay. (**b**) Simulation results of Magnitude and phase imbalance for the proposed WPD.

**Figure 17:**
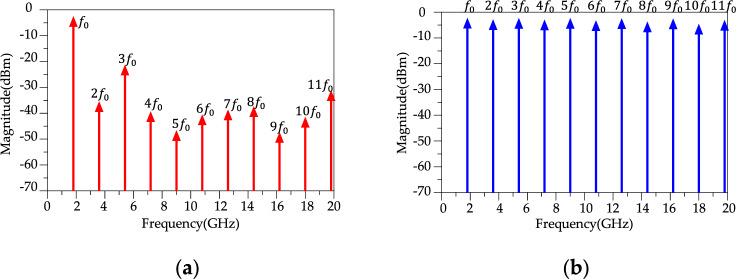
Output harmonics. (**a**) Proposed modified WPD. (**b**) Conventional WPD.

According to Fig. 15b, the simulated results of the proposed modified WPD illustrate an ultra-wide stopband up to 40 GHz with a 20 dB attenuation level. According to simulated results in Fig. 15b, above 20 GHz, the *S*_11_ loss increases which can be attributed to several high frequency loss sources such as material losses (dielectric losses, conductor losses and parasitic components), impedance mismatches and radiation losses from transmission lines and components. Fig. 16 shows the imbalance EM analysis that depicts the simulation results of the group delay, phase difference and magnitude difference between the two outputs. According to the results, the group delay, phase and magnitude imbalance are better than 0.5 ns ,0.15 degrees and 0.00018 at *f* = 1.8 GHz, respectively, this discrepancy in output power levels can be attributed to simulation inaccuracies and a slight variation of 0.001 mm in the overall design. It could also arise from an asymmetrical positioning with a modeling difference of approximately 0.001 mm. Fig. 17 shows the simulated results of harmonic balance analysis (with *P*_IN_ = 0 dBm at *f* = 1.8 GHz) for the conventional WPD and the proposed modified WPD. To simulate the harmonics, the harmonic balance simulation in ADS software was used. It is important to note that even in passive devices like Wilkinson Power Dividers (WPDs), the generation of undesired harmonics can occur for various reasons. These reasons include impedance mismatches at the input or output ports, the presence of non-ideal components (such as non-linear parasitic capacitance and inductance in high-frequency resistance models), and crosstalk between different branches, which can result in signal reflections within the WPD and the subsequent generation of unwanted harmonics. The fundamental frequency (*f* = *f*_0_) is located in the passband of the proposed LPF structure, therefore, the output magnitude remains constant and equals 3 dBm for both conventional and modified WPDs. According to Fig. 17a, other harmonics (2nd to 11th) are located in the stopband of the LPF structure that are suppressed significantly.

## Results and discussion

**Figure 18:**
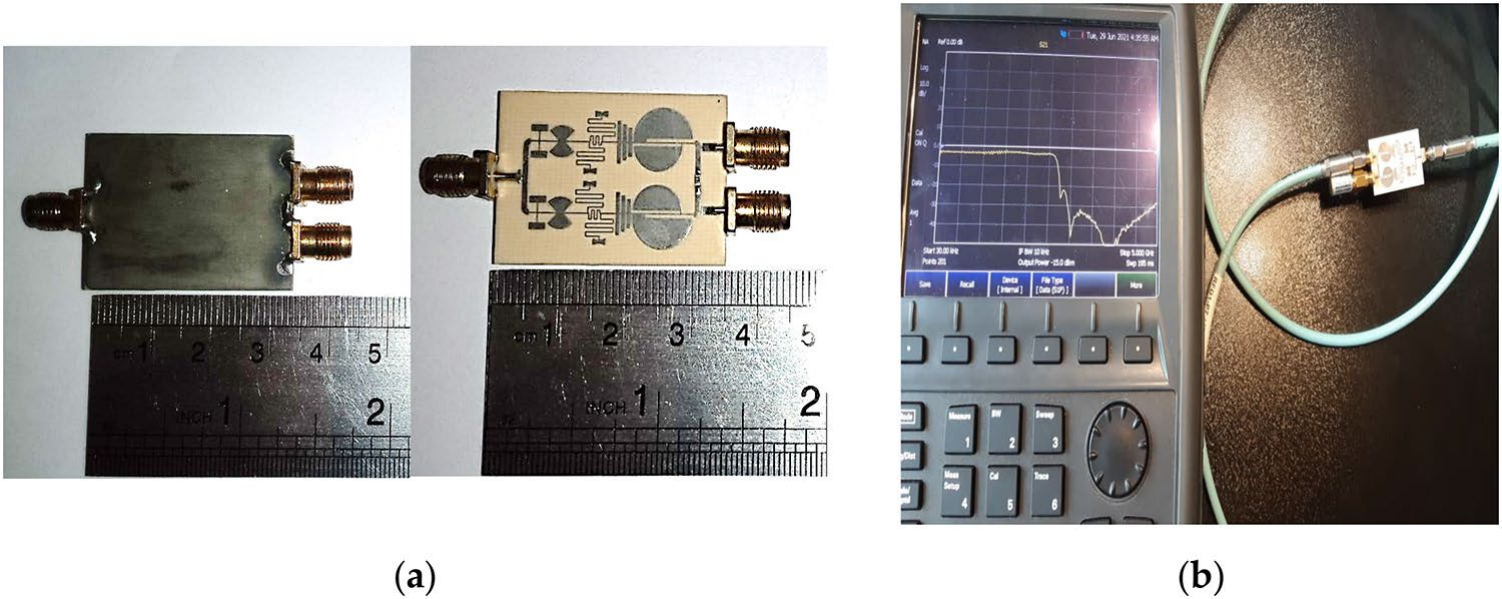
Fabricated sample of modified WPD (**a**) Top/bottom views. (**b**) Experimental setup.

**Figure 19:**
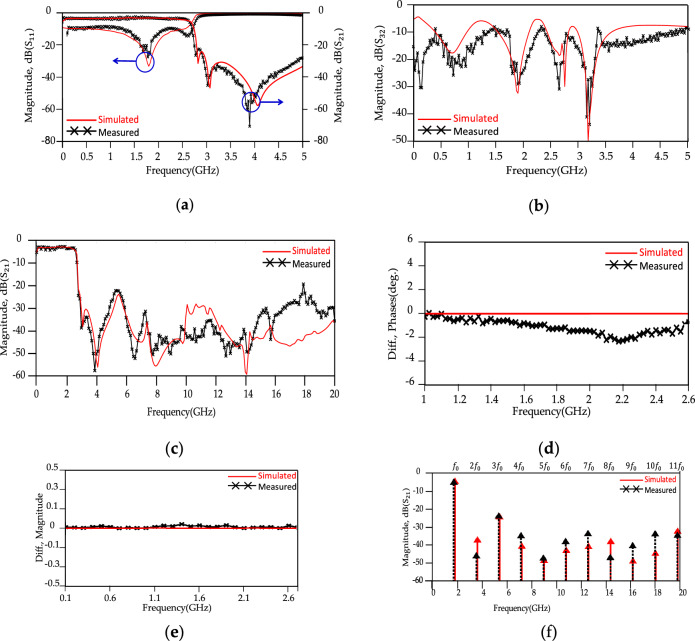
The proposed WPD simulated and measured results. (**a**) S_21_ parameter from 0 to 5 GHz, (**b**) S_23_ parameter from 0 to 5 GHz, (**c**) S_21_ parameter measured from 0 to 20 GHz, (**d**) Phase difference, (**e**) Magnitude difference,(**f**) Output harmonics (harmonic balance).

The proposed modified WPD is implemented on the Rogers RO4003c substrate with a dielectric constant of 3.38, a thickness of 0.8 mm, and a loss tangent of 0.0022. An AGILENT/HP N9918A Vector Network Analyzer was used to measure the S-parameters response with a frequency range of 0.1–20 GHz. The fabricated sample of the presented modified WPD and experimental/measurement setup are shown in Fig. 18. The simulated and measured results are shown in Fig. 19. According to Fig. 19d,e, the reasons for the structural imbalance include manufacturing and assembly errors of SMA connectors, as well as measurement errors by VNA and cables.

**Figure 20:**
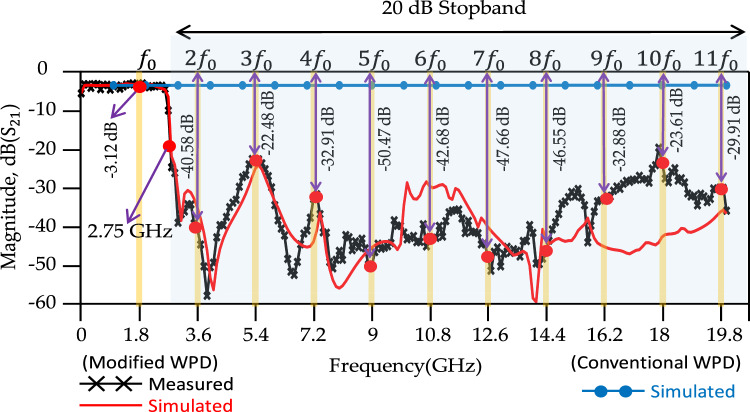
Harmonic suppression, simulation and measurement results of proposed conventional and modified WPDs.

According to the measured results, the isolation between output ports (|S_32_|), insertion loss (|S_21_) and input return loss (|S_11_|) are better than 21.2 dB, 3.52 dB and 33.2 dB, respectively, at *f* = 1.8 GHz. There is a good agreement between simulated and measured results. According to the results, the proposed WPD shows a wide stopband from 2.7 to 40 GHz and 2.7 to 20 GHz with 20 dB attenuation levels for both the simulated and measured results, respectively. Figure 20 shows the stopband bandwidth and harmonic suppression level for the proposed modified WPD. The second to eleventh harmonics at frequencies 2*f*_0_ to 11*f*_0_ (*f*_0_ = 1.8 GHz) are depicted to be suppressed with better than −40.85, −22.48, −32.91, −50.47, −42.68, −47.66, −46.55, −32.88, −23.61 and −29.91 (all in dB) attenuation levels. Compared with the state-of-the-art WPDs, desirable suppression levels in the stopband are illustrated for the modified WPD and harmonics have been significantly attenuated in this region. Table 2 shows a comparison between the obtained results for the proposed modified WPD and other works described in the literature.

**Table 2:** Comparison of the proposed modified WPD with related previous works.

Refs	${f}_{0}$ (GHz)	${S}_{32}$ (dB)	${S}_{11}$ (dB)	${S}_{21}$ (dB)	${S}_{22}$ (dB)	20 dB Harmonics suppression	Size (λ^2^)	20 dB Stopband (GHz)
^26^	1.9	26	30	4	–	2nd–4th	0.1 × 0.07	4.1–7.6
^27^	1.8	17	17	3.5	20	2nd–4th	0.25 × 0.15	2.9–7.4
^28^	2	20	21	3.3	26	2nd–3rd	0.32 × 0.24	–
^29^	2.65	22	27	3.4	–	3rd & 5th	–	–
^30^	0.9	–	36	3.32	–	3rd	–	–
^31^	1	30	30	3.2	–	2nd–3rd	–	2.2–4
^32^	4.5	10	12	3.3	12	2nd–6th	0.23 × 0.41	–
^33^	0.5	39	26	3.1	26	No	–	–
^35^	1.8	20.1	21.2	3.1	–	2nd–11th	–	4.4–20
^37^	1.8	34	20.4	3.1	24.6	2nd–6th	–	3.4–10.9
^38^	1	13.5	30	3.4	–	2nd–5th	–	–
^39^	0.9	23	20	4.75	20	No	0.1 × 0.2	–
^40^	1.5	23.8	19.9	3.2	–	2nd–5th	–	3.3–7.9
^41^	1.8	15.2	16.5	4	14.3	No	0.36 × 0.02	–
^44^	1.8	31.2	34.2	3.52	26.2	2nd–7th	0.42 × 0.33	2.54–13.48
This work Meas	1.8	21.2	33.2	3.23	–	2nd–11th	0.38 × 0.27	2.7–20

Among power dividers, WPDs are widely used due to their simple construction, narrow bandwidth, and reliable performance. WPDs can be used in test systems to measure two different characteristics of a signal, such as frequency and power, for broadband-independent signal sampling. GSM modems have a wide range of applications in transaction terminals, supply chain management, and security applications. One of the frequently used frequency bands in GSM is the 1800 MHz range, known for its widespread availability and well-established presence within GSM networks^44^. The proposed WPD shows desirable performance at this frequency, in terms of having a wide stop band, high isolation, and suppression of unwanted harmonics. The proposed WPD is versatile and finds application in various scenarios, including distributing power to antennas in an array, partitioning power among different components within a system, and aggregating power from various system segments. Moreover, the presented WPD is suitable for use in devices like radio receivers and transmitters, as well as more advanced systems found in the telecommunications and commercial sectors.

## Conclusions

This paper presented an efficient WPD with LPF harmonic suppression structure and ultra-wide stopband for modern communications. An LPF structure was designed based on EM/Microwave theoretical equations. The proposed LPF structure was integrated into both branches of the WPD to improve the stopband, isolation and harmonics. The proposed modified WPD demonstrated a desirable performance for the S parameters at *f* = 1.8 GHz. The demonstrated results showed an RL, IL and isolation better than 33.2 dB, 3.23 dB and 21.2 dB, respectively. The proposed modified WPD was able to suppress the 2nd to 11th harmonics with better than 20 dB attenuation level and showed an ultra-wide stopband. The overall dimensions of the structure are 35 mm × 2 5 mm. The proposed WPD is, therefore, suitable for a wide range of cutting-edge wireless communication systems in GSM and LTE services. The proposed power divider can be used to feed antennas in an array, divide power between different parts of a system, or collect power from different parts of a system. Also, the proposed power divider can be used in different structures such as radio receivers and transmitters and more advanced systems in the telecommunications and commercial industries.

## Data Availability

The corresponding author can be contacted on reasonable request.
